# Cryobiopsy as a Diagnostic Tool in Lung Transplantation: A Case Report

**DOI:** 10.7759/cureus.68554

**Published:** 2024-09-03

**Authors:** Jhon Edwar Garcia Rueda, Juan David Botero Bahamón, Alejandro Cardona Palacio, Felipe de Jesus Campo Campo, Maria Isabel Palacio Mejía

**Affiliations:** 1 Internal Medicine, Pontifical Bolivarian University, Medellín, COL; 2 Pulmonology, Cardio Clinical VID, Medellín, COL; 3 Pathology, Pablo Tobón Uribe Hospital, Medellín, COL; 4 Epidemiology, Cardio Clinical VID, Medellín, COL

**Keywords:** graft rejection, transplant biopsy, post-lung transplant complications, lung biopsy, lung surgery

## Abstract

Lung transplantation is an option for patients with advanced lung pathologies. Transbronchial biopsies are routinely conducted during the first year to manage acute rejection episodes, with chronic rejection, particularly bronchiolitis obliterans syndrome, becoming a significant concern thereafter. A 34-year-old patient with a diagnosis of primary ciliary dyskinesia was admitted to the emergency room due to a severe exacerbation that caused mixed respiratory failure. He required intubation and extracorporeal membrane oxygenation (ECMO) support as a bridge to bilateral lung transplantation. Post-transplantation, cryobiopsy was implemented according to local protocol, revealing A3 rejection without microbiological isolations. The implementation of cryobiopsy in lung transplantation proves to be an effective diagnostic strategy, offering enhanced tissue evaluation and improved diagnostic performance in both acute and chronic cellular rejection.

## Introduction

Lung transplantation is an option for patients with advanced lung pathologies whose survival is expected to be less than 50% in the next two years [1]. For the period between 2010 and 2018, there are reports of 33,891 transplants by the International Society for Heart and Lung Transplantation (ISHLT), with idiopathic pulmonary fibrosis (IPF) being the primary indication, followed by chronic obstructive pulmonary disease (COPD). Beyond the first post-transplant year, during which the main cause of death is derived from infectious pathologies, chronic graft dysfunction, predominantly represented by bronchiolitis obliterans syndrome (BOS), becomes the leading cause of death [2,3].

To mitigate the effects of acute rejection, transplant centers routinely perform transbronchial biopsies (TBBs), mainly during the first year, to address unnoticed episodes as well as in the context of suspected rejection [4,5]. Transbronchial cryobiopsy (TBCB) is a diagnostic strategy that employs a freezing probe to obtain a larger sample of lung tissue while preserving its structure. This approach has only recently been utilized to enhance the diagnostic performance of rejection in lung transplantation.

Proper evaluation of lung tissue is crucial for the diagnosis and management of complications in patients undergoing lung transplantation. Traditionally, transbronchial forceps biopsies (TBFB) have been considered the gold standard for detecting acute allograft rejection, infections, and other post-transplant pathologies [6]. However, this method has significant limitations, such as the small size of the samples obtained and a high incidence of complications, including bleeding and pneumothorax, reported in up to 5% of cases [7].

In response to these limitations, TBCB has emerged as a promising alternative technique. Unlike forceps biopsies, cryobiopsy uses a flexible cryoprobe that freezes the lung tissue, allowing for the extraction of larger samples with better structural preservation, which facilitates a more accurate and detailed diagnosis [8]. Recent studies have shown that samples obtained by TBCB are significantly larger and contain a higher percentage of alveolar tissue compared to those obtained by traditional methods, thereby improving diagnostic yield [2].

In addition to the superiority in sample quality, TBCB has shown a favorable safety profile. In a comparative study, cryobiopsy resulted in lower rates of complications such as bleeding and pneumothorax and reduced procedure time compared to TBFB, which also reduced radiation exposure for patients and staff [2,9].

Given these advantages, cryopbiopsy could represent a significant advancement in post-transplant monitoring, improving both diagnostic accuracy and patient safety. To the best of our knowledge, the following describes the experience of the first reported case in Colombia in which this technique was employed as a diagnostic method for acute or chronic rejection.

## Case presentation

A 34-year-old man with a diagnosis of primary ciliary dyskinesia was on the waiting list for transplantation. He was admitted to the emergency room due to a severe exacerbation that caused mixed respiratory failure, for which intubation and extracorporeal membrane oxygenation (ECMO) bridging to transplantation were provided.

**Figure 1:**
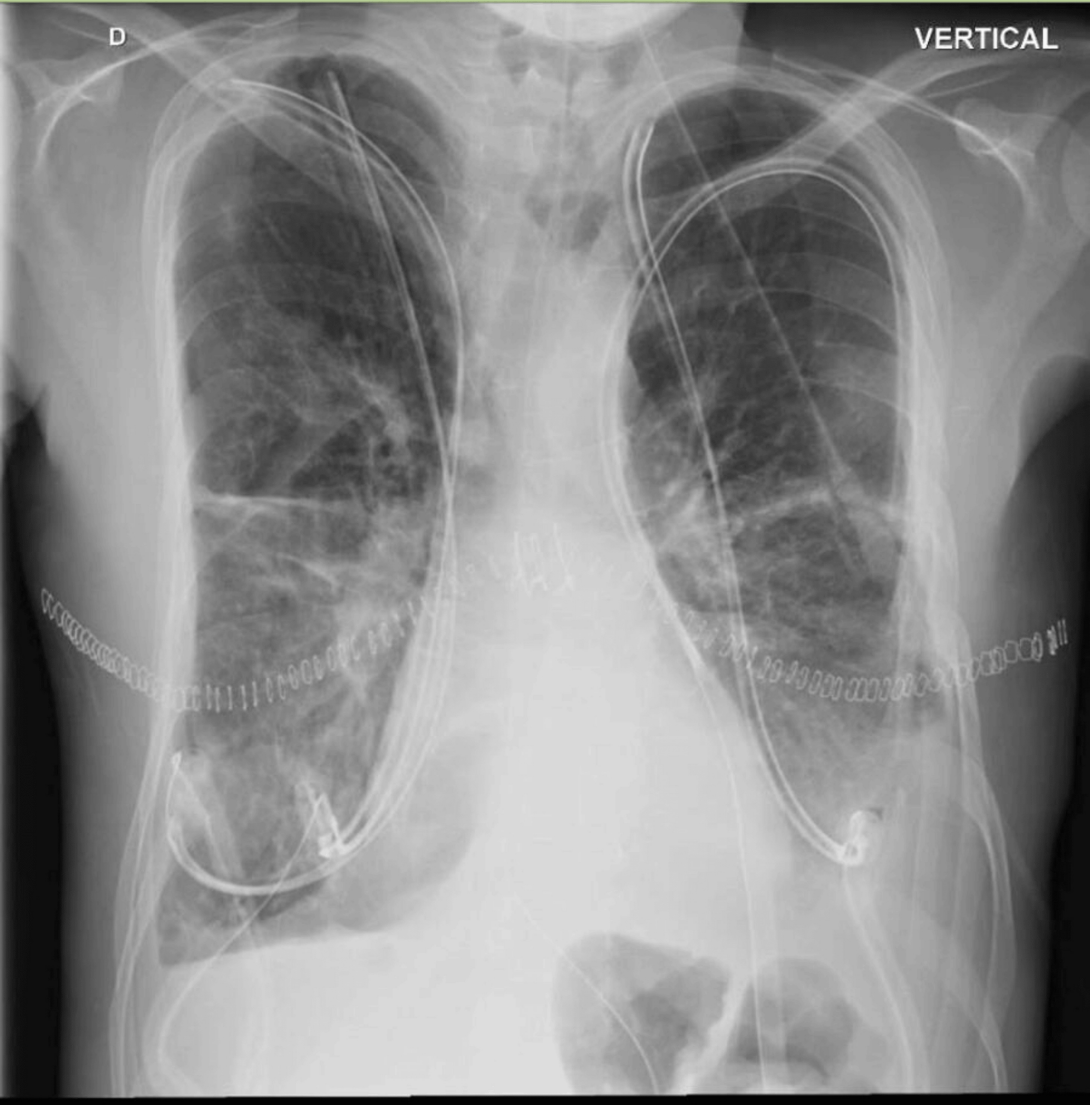
Chest X-ray

During his stay in the intensive care unit (ICU), the patient's clinical evolution was favorable, for which reason he underwent a tracheostomy while maintaining ECMO support and was also provided with rehabilitation and nutrition (Figure 1).

After three months of ECMO support, the patient was taken for a bilateral lung transplant, with adequate evolution; tracheostomy decannulation was achieved, and the chest tubes were removed without pleural effusion, although with initial dysphagia, for which reason some medications and intravenous cyclosporine were given through the nasogastric tube. During follow-up, he presented slightly decreased levels of cyclosporine concentrations, and images showed poorly defined centrilobular and alveolar nodules.

**Figure 2:**
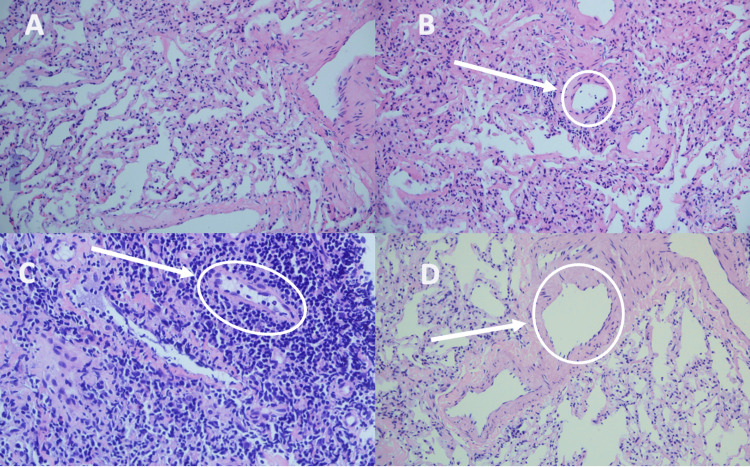
Lung cryobiopsy (A) Acute cellular rejection, grade A3; (B) arrow shows focal areas of capillaritis; (C) arrow shows peribronchovascular inflammatory infiltrate; (D) histologic improvement and resolution of acute cellular rejection, with the arrow showing the absence of infiltrate.

At the time of transplantation, the local protocol had established cryobiopsy follow-up for the first month, six months, and one year after transplantation. Cryobiopsy was performed in the first month after transplantation and involvement was documented due to A3 rejection without microbiological isolations (Figures 2A-2B); it was complemented with CD4 staining and humoral rejection was ruled out. By this time, swallowing problems had been appropriately optimized, so intravenous boluses of methylprednisolone (500mg) were given for three days with subsequent high-dose corticosteroids (1 mg/kg), and calcineurin inhibitor rotation (reaching serum levels rapidly with immediate-release tacrolimus (15 ng/mL)) was also performed. With this strategy, control of rejection was achieved in the follow-up cryobiopsy at three weeks (Figures 2C-2D), in addition to the resolution of the symptoms. One year later of the follow-up, he remains asymptomatic with the following treatment regimen: extended-release tacrolimus 20mg every 24 hours, everolimus 1mg every 12 hours, prednisone 5mg every 24 hours.

## Discussion

Acute rejection after the first year of transplantation can present asymptomatically or with minimal symptoms, posing a challenge for timely diagnosis and management [10]. Recently, cryobiopsy has gained attention in post-transplant monitoring due to its superior diagnostic capabilities. Compared to standard TBB using forceps in ventilated patients with clinical signs of rejection, cryobiopsy demonstrated a diagnostic yield of 96.6%, significantly higher than the forceps method. Moreover, cryobiopsy detected 24% more cases of rejection in follow-up biopsies and identified more severe forms of rejection when used alongside standard TBBs [11,12].

TBCB has emerged as a superior technique for diagnosing diffuse interstitial lung diseases (ILDs) compared to TBFB. Cryobiopsy provides significantly larger tissue samples, with an average area of 43.11 mm² compared to the 3.3 ± 4.1 mm² obtained via forceps-TBB. This difference in sample size is crucial for accurate diagnosis, as the samples obtained by TBCB exhibit better preservation of tissue architecture, with fewer crushing and hemorrhage artifacts than those seen in TBFB. This improves the quality of histopathological analysis and allows for the successful application of immunohistochemistry techniques [13]. Despite these benefits, TBCB has a higher risk of pneumothorax, with an incidence of 9.5% compared to 6% in forceps biopsies. However, there were no significant differences in hemorrhagic complications between the two techniques (23.7% in TBCB versus 20.8% in TBFB, p > 0.05) [14].

The application of cryobiopsy in lung transplantation enhances the evaluation of lung parenchyma, allowing for a more comprehensive assessment. This technique expands the evaluable area from 0.03 cm^3^, containing 2,652 alveoli without bronchioles in a standard TBB, to 0.65 cm^3^, containing 9,956 alveoli with up to two bronchioles in cryobiopsy; this technique significantly improves diagnostic accuracy in both acute and chronic cellular rejection [15]. Furthermore, this comprehensive sampling supports the standardization of lung transplant patient evaluation, aligning with ISHLT guidelines, which classify pulmonary rejection by severity: Grade A for acute rejection, Grade B for airway inflammation, Grade C for chronic rejection, and Grade D for chronic vascular rejection [16].

In our initial experience, cryobiopsy demonstrated excellent diagnostic performance, particularly in a high-risk patient (prolonged critical condition and suboptimal immunosuppression due to dysphagia). It represents the first experience of implementing cryobiopsy in transplantation within our setting. This case highlights the potential of cryobiopsy to improve the management of acute rejection episodes in lung transplant patients. Future studies are needed to assess whether this early diagnostic capability will significantly influence the development of BOS and impact graft survival, especially given the evolving management strategies that have achieved 10-year survival rates in some cases [17].

## Conclusions

The implementation of cryobiopsy in lung transplantation represents an effective diagnostic strategy for evaluating lung transplant patients, offering enhanced diagnostic accuracy for both acute and chronic cellular rejection. Our initial experience suggests that cryobiopsy could help standardize the evaluation of these patients. However, its adoption should be carefully considered based on individual risks, the medical team's expertise, and the patient's specific needs. Future research should focus on optimizing safety protocols and precisely defining clinical indications to ensure that the benefits of TBCB outweigh the potential risks in each clinical context.
